# Parental psychopathology and expressed emotion in children with avoidant/restrictive food intake disorder

**DOI:** 10.1186/s13034-025-00929-x

**Published:** 2025-06-06

**Authors:** Hannah Lea Klüber, Annick Martin, Franziska Schlensog-Schuster, Andreas Hiemisch, Wieland Kiess, Anja Hilbert, Ricarda Schmidt

**Affiliations:** 1 Integrated Research and Treatment Center (IFB) AdiposityDiseases, Behavioral Medicine Research Unit, Department of Psychosomatic Medicine and Psychotherapy, Leipzig University Medical Center, Philipp-Rosenthal-Straße 55, 04103 Leipzig, Germany; 2 Department of Child and Adolescent Psychiatry, Psychotherapy and Psychosomatics, Leipzig University Medical Center, Liebigstrasse 20a, 04103 Leipzig, Germany; 3 University Hospital of Child and Adolescent Psychiatry and Psychotherapy, University of Bern, Bern, Switzerland; 4 LIFE Leipzig Research Center for Civilization Diseases, Leipzig University, Philipp-Rosenthal-Strasse 27, 04103 Leipzig, Germany; 5 Hospital for Children and Adolescents, Center for Pediatric Research, Leipzig University Medical Center, Liebigstrasse 20a, 04103 Leipzig, Germany; 6 German Center for Child and Adolescent Health (DZKJ), partner site Leipzig/Dresden, Leipzig, Germany

**Keywords:** Eating disorders, ARFID, Expressed emotion, Parents, Family factors

## Abstract

**Background:**

Family factors like parental psychopathology and parental expressed emotion, referring to the emotional atmosphere within a family, play a significant role in the maintenance and treatment outcome of anorexia nervosa. However, nothing is known about these parental characteristics in avoidant/restrictive food intake disorder (ARFID).

**Objective:**

This study aimed to determine the proportion of parents exceeding clinical cutoffs for depression, eating disorder psychopathology, and expressed emotion, specifically criticism and emotional overinvolvement, in ARFID, anorexia nervosa (AN), and healthy controls (HC), and to evaluate group differences. Associations between parental characteristics and child illness characteristics were analyzed.

**Method:**

Treatment-seeking children and adolescents (0–17 years) with ARFID (*n* = 42) were compared to those with AN (*n* = 25) and HC (*n* = 42) in parental eating disorder psychopathology (Eating Disorder Examination-Questionnaire 8), parental depression (Patient Health Questionnaire-9), and parental expressed emotion (Family Questionnaire).

**Results:**

When comparing ARFID with AN and HC, the proportions of parents exceeding clinical cutoffs for depression (26% vs. 20% vs. 14%), eating disorder psychopathology (7% vs. 12% vs. 9%), and criticism (26% vs. 32% vs. 29%) did not differ significantly. For emotional overinvolvement (41% vs. 52% vs. 0%), differences emerged between ARFID and HC, but not AN. Dimensionally, levels of parental depressive symptoms and emotional overinvolvement were higher in those with ARFID versus HC only. More parental depressive symptoms, criticism, and emotional overinvolvement were significantly related to greater children’s restrictive eating behaviors, lower standardized body-mass-index, and lower number of accepted foods.

**Conclusion:**

ARFID and anorexia nervosa were found to share similar distributions in parental psychopathology and parental expressed emotion. Future studies may focus on the role of those family factors in development and outcome of ARFID.

**Supplementary Information:**

The online version contains supplementary material available at 10.1186/s13034-025-00929-x.

## Introduction

Avoidant/restrictive food intake disorder (ARFID) is characterized by inadequate nutritional and/or energy supply leading to significant physical and/or psychosocial impairment [1, 2]. Unlike anorexia nervosa (AN) [2], a restrictive eating disorder as well, body image disturbance and weight-loss intentions are not the main drivers for limited food intake in ARFID. Epidemiologically, ARFID tends to present with earlier onset, a more prolonged symptom duration in patients, and a higher prevalence among males than AN, and frequently co-ocurs with physical or mental comorbidities [3, 4]. To date, genetic predispositions [5, 6] in interaction with biological and psychological factors (sensory perception, homeostatic appetite, fear of aversive consequences) [7] have been discussed as essential in the onset and maintenance of ARFID. Still, little is known about the disorder from an interpersonal and familial perspective [8, 9].

Family factors, like parental psychopathology and parental expressed emotion, have been identified as maintenance factors in feeding and eating disorders, specifically in AN [see 10–15]. Expressed emotion describes the emotional climate within a family, particularly toward a relative with a mental disorder [10]. It is a multidimensional construct comprising five components: critical comments, reflecting expressions of disapproval; emotional overinvolvement (EOI), encompassing excessive concern or overprotectiveness; hostility, indicating rejection or generalized negative attitudes; warmth, referring to displays of empathy and affection; and positive remarks, which capture affirming or supportive statements. High levels of expressed emotion, particularly in the form of criticism, hostility, and EOI, have been associated with unfavorable clinical outcomes across various psychiatric conditions, including eating disorders [16–18]. Studies in families with an adolescent with AN revealed higher parental expressed emotion compared to healthy control families [19–21]. Higher expressed emotion in families having a child with AN was related to higher age, longer illness duration [19], higher eating disorder psychopathology and lower standardized body mass index (BMI, kg/m²) in children at baseline [10, 23, 24], and higher relapse rates and poorer treatment outcome [15, 24–26]. Nevertheless, it remains unclear whether elevated levels of parental expressed emotion are caused by the children’s illness or the other way around, pointing out a relational perspective on the matter [27, 28].

Regarding parental psychopathology, around 13% of parents having a child with AN exceeded clinical cutoff for depressive symptoms [29], presenting with higher mean levels of depressive symptoms than parents of children without an eating disorder [30, 31]. Similarly, Cerniglia et al. [32] identified more depressive symptoms and hostility in mothers of children aged 2–3 years with ARFID compared to mothers of children without feeding disorder. In children aged < 3 years with infantile anorexia [33], which may be considered an early presentation of ARFID, and children aged 2–10 years with ARFID symptoms, it was found that parental psychopathology, particularly disordered eating behaviors (e.g., dieting, bulimia, food preoccupation, oral control) and clinically significant depressive symptoms, were positively associated with children’s feeding problems, dysfunctional dyadic feeding interactions, and greater malnutrition severity over time [34–38]. Relative to mothers of healthy controls, those having a 0–3-year-old child with feeding difficulties related to signs of malnutrition, problematic parent-child feeding interaction and food avoidance behaviors reported more disordered eating behaviors, including restraint, eating concern, shape concern and weight concern, as well as depressive symptoms [34, 39–41]. From a transactional perspective, these characteristics were found to contribute to the development and maintenance of feeding difficulties via poor feeding interactions or parenting [14, 42].

Considering that parental factors play a crucial role in feeding disorders and eating disorders, while evidence for ARFID scarce so far, the aim of this study was (1) to determine the proportion of parents exceeding clinical cutoffs for eating disorder psychopathology, specifically restraint, eating concern, weight concern and shape concern, depressive symptoms, and parental expressed emotion, specifically, critical comments and emotional overinvolvement, in families having a child with ARFID, and (2) to compare these characteristics to those of families without children with feeding disorder or eating disorder and families having a child with AN. (3) It was aimed to examine associations between these parental factors and children’s characteristics in the whole sample. We hypothesized that children’s symptomatology, specifically food avoidance behaviors, showed positive associations with parental psychopathology and parental expressed emotion, while the number of accepted foods and children’s standardized BMI showed negative associations with parental psychopathology and parental expressed emotion.

## Methods

### Participants

The data for this study were derived from the validation study of the ARFID module for the Eating Disorder Examination [43] including treatment seeking and non-treatment-seeking participants recruited between February 2018 and October 2021. Recruitment of the clinical groups took place via the Eating and Feeding Disorder Unit of Leipzig Medical Center, Germany, a support group for patients with ARFID, and Internet advertisement, while the control group was primarily recruited via the population-based LIFE Child study [44], which longitudinally investigates child development and health. Inclusion criteria comprised child age between 0 and 17 years as well as the presence (clinical groups) or absence (control group) of restrictive eating disorders. No specific exclusion criteria in addition to sufficient German language skills were applied. Children ≥ 8 years and at least one parent gave written informed assent and consent prior to study inclusion. The study was approved by the Ethics Committee of the Medical Faculty of the University of Leipzig (Reg. No. 264-10-19042010). A financial compensation of 15€ was offered for participation.

For the present study, only children and adolescents with an interview-based diagnosis of ARFID and AN, as well as controls were included. Controls were individually matched to those with ARFID based on age and sex. For diagnosis, the full-length EDE [45, 46], its child adapted version (ChEDE) [47, 48], and the EDE ARFID module [43] were used. In addition to current diagnostic criteria covering the past 3 months, all diagnostic items were asked for the time before the past 3 months in case that ARFID or AN was not present currently due to positive effects of treatment, but as a past diagnosis (at the time of treatment start). Out of 185 eligible families from the original study sample, *n* = 50 patients had a diagnosis of ARFID, of which *n* = 42 patients with ARFID provided data on parental measures. All of the *n* = 25 patients with AN from the original sample were included.

### Assessments

#### Parental characteristics

**Eating Disorder Psychopathology.** The 8-item self-report Eating Disorder Examination-Questionnaire 8 (EDE-Q8) [49] covers restraint, eating concern, weight concern, and shape concern, with items being rated on a 7-point Likert-scale ranging from 0 (feature was not present) to 6 (feature was present every day or in extreme form) referring to the past 28 days. A global mean score ≥ 90 th percentile indicates a risk for clinically relevant eating disorder psychopathology [49]. The present study yielded Cronbach’s Alpha of 0.91 suggesting excellent internal consistency.

**Depressive Symptoms.** The 9-item Patient Health Questionnaire-9 (PHQ-9) [50; German validation 51] covers DSM-5 criteria for depression, with items being rated on a 4-point Likert-scale ranging from 0 (not at all) to 3 (nearly every day) referring to the past 2 weeks. A sum score ≥ 10 indicates a risk for major depression [50]. The present study found Cronbach’s Alpha of 0.79 suggesting acceptable internal consistency.

**Expressed Emotion.** The 20-item Family Questionnaire (FQ) [52] was applied using its subscale scores for critical comments (CC) and emotional overinvolvement (EOI). All items were rated on a 4-point Likert-scale ranging from 0 (never/very rarely) to 4 (very often). A cut-off of ≥ 23 in CC and a cut-off of ≥ 27 in EOI indicate the risk for high Expressed Emotion (EE) [51]. The present study yielded Cronbach’s Alpha of 0.88 for the subscale of CC and a Cronbach’s Alpha of 0.86 for the subscale of EOI suggesting good internal consistency.

#### Children’s characteristics

**Standardized BMI.** The BMI standard deviation score (BMI-SDS) based on objectively measured weight and height was extracted from patient’s medical record. Based on age and sex, a BMI-SDS of 0.0 corresponds to a BMI percentile of 50 indicating normal weight, while BMI-SDS scores of +/−1.28 correspond to a BMI percentile of 90/10 indicating overweight or underweight, respectively.

**Child Eating Behavior Questionnaire.** The Child Eating Behavior Questionnaire (CEBQ) [53, German translation Ulbrich et al., in preparation] was applied to parents for assessing child food avoidance behaviors, specifically, satiety responsiveness, slowness in eating, emotional undereating, and food fussiness. All items were rated on a 5-point Likert scale ranging from 1 (never) to 5 (always) which yielded mean subscale scores. CEBQ’s internal reliability for the present study found a Cronbach’s Alpha between 0.71 and 0.93 suggesting acceptable to excellent internal consistency. The CEBQ has previously been applied in samples with ARFID [54, 55], demonstrating good reliability, discriminant validity between children with ARFID and controls, and convergent validity with measures of ARFID symptoms.

**Food List.** Children’s food variety was measured via the number of accepted foods assessed by a food list [unpublished Sarah Eckhardt]. On a list including 14 food categories and one category for drinks, children older than 13 years and parents of children aged 0–13 years provided information about accepted foods of the child. The number of accepted foods per food group was extracted, with a maximum of 40 foods for meat, sausage, and fish, 38 for fruits, 36 for carbohydrates and bakery products, 34 for vegetables, 24 for sweets and snacks, and 14 for potato products and meals. Higher sum scores—ranging between 0 and 235—indicated a greater food variety. The food list demonstrated good discriminant validity in distinguishing between ARFID and AN and convergent validity with measures on ARFID symptoms [56].

All parent- and child-related questionnaires have been validated for the use in German.

### Data analytic plan

For study aim (1), a descriptive data analysis was performed on the number of parents exceeding clinical cutoffs for the EDE-Q8 global mean score, PHQ-9 sum score, and FQ subscale scores for CC and EOI per diagnosis groups (ARFID, AN, HC). In order to test whether clinically relevant psychopathology and EE were differentially distributed among groups, χ²-tests with Bonferroni corrections were conducted and Cramer’s *V* was reported for effect size estimation, which can be interpreted as small (0.10), medium (0.30), or large (0.50).

For hypothesis (2), repeated measure analysis of variances (rmANOVA) was performed in order to compare parental psychopathology (EDE-Q8 global score, PHQ-9 sum score) and expressed emotion (FQ subscale score for CC and EOI) between ARFID and HC group due to the individually matched design of these groups. In contrast, for comparisons between ARFID and children with AN, where no matching was applied, multivariate analyses of covariance (MANCOVA) were used to adjust for significant differences in age and sex. In terms of violation of normality and homogeneity of variances, non-parametric tests were conducted but only reported if deviating from parametric test results. Effect sizes for between-group differences were estimated with partial η² which can be interpreted as small (0.01), medium (0.06), or large (≥ 0.14).

For hypothesis (3), Pearson correlation analyses were conducted to examine associations between parental psychopathology (EDE-Q8 global score, PHQ sum score) and expressed emotion (FQ subscale scores for CC and EOI) and children’s illness characteristics (BMI-SDS, number of accepted foods, and CEBQ subscale scores). Effect size *r* was interpreted as small (0.10), medium (0.30) and large (0.50). All statistical analyses were performed using IBM^®^ SPSS Statistics ^®^ version 29.0, using a two-tailed α < 0.05.

## Results

### Sample characteristics

Demographic characteristics of the sample are delineated in Table 1. Patients with ARFID and individually matched HC were approximately 7 years old and relatively balanced for sex (45.0% female in ARFID; 47.6% female in HC, considering that a 13-month-old boy with ARFID could only be matched to a 9-month-old girl in the HC group). Those with AN were significantly older and more often female, *p* <.05. The total sample was homogeneous in terms of nationality without group differences. While children with ARFID and AN did not differ significantly in BMI-SDS, HC had a significantly higher BMI-SDS and were more often classified as normal weight, *p* <.05.

**Table 1:** Demographic characteristics of children and their parents per group

	ARFID (*n* = 42)	HC (*n* = 42)	AN (*n* = 25)
	*M ± SD*	*M ± SD*	*M ± SD*
Child age, years	7.33 ± 5.39	7.31 ± 5.29	14.80 ± 1.61
Child BMI-SDS	−1.55 ± 1.04	−0.38 ± 0.93	−1.37 ± 1.09
Parental age, years	37.15 ± 6.83	39.48 ± 6.36	47.36 ± 5.11
Parental BMI, kg/m²	24.82 ± 6.42	23.68 ± 4.16	24.41 ± 4.41
	*n* (%)	*n* (%)	*n* (%)
Child sex, female	19 (45.2%)	20 (47.6%)	24 (96.0%)
Child nationality, German	42 (100.0%)	41 (97.6%)	42 (100.0%)
Child weight status			
Severe underweight (BMI-SDS < −1.88 kg/m²)	20 (47.6%)	2 (4.8%)	8 (32.0%)
Normal weight (−1.28 ≤ BMI-SDS < 1.28 kg/m²)	15 (35.7%)	34 (81.0%)	14 (56.0%)
Overweight (BMI-SDS ≥ 1.28 kg/m²)	0	1 (2.4%)	0
Parental sex, female	38 (90.5%)	41(97.6%)	20 (80.0%)
Parental weight status			
Underweight (BMI < 18.5 kg/m²)	3 (7.1%)	1 (2.4%)	1 (4.0%)
Normal weight (18.5 ≤ BMI < 25.0 kg/m²)	23 (54.8%)	27 (64.3%)	14 (56.0%)
Overweight (25.0 ≤ BMI < 30.0 kg/m²)	8 (19.0%)	10 (23.8%)	7 (28.0%)
Obesity (BMI ≥ 30.0 kg/m²)	7 (16.7%)	3 (7.1%)	2 (8.0%)
Parental education			
Low education (< 12 school years)	21 (50.0%)	9 (21.4%)	10 (40.0%)
High education (≥ 12 school years)	20 (47.6%)	33 (78.6%)	15 (60.0%)

*ARFID*  Avoidant/restrictive food intake disorder,* HC*  healthy controls,* AN* Anorexia Nervosa,* BMI* body mass index,* SDS* standard deviation score. Values may not sum up to 100% due to missing values

### Parental psychopathology and expressed emotion

The number of parents of children with ARFID, AN, and HC exceeding clinical cutoffs in ED psychopathology, depressive symptoms, critical comments, and emotional overinvolvement can be seen in Table 2. Except for emotional overinvolvement (*p* <.001), no association between parental characteristics with group status was found. Post-hoc Bonferroni tests showed that the ARFID and AN groups more often exceeded the cutoff for emotional overinvolvement than HC, with large effects, without significant differences between each other.

**Table 2:** The proportion of parents exceeding clinical Cut-offs for psychopathology and expressed emotion per group

	ARFID (*n* = 42)	HC (*n* = 42)	AN (*n* = 25)	Test		
	*n* (%)	*n* (%)	*n* (%)	χ²	*p*	*V*
EDE-Q8	3 (7.1)	4 (9.4)	3 (12.0)	0.45	0.797	0.065
PHQ-9	11 (26.2)	6 (14.3)	5 (20.0)	1.85	0.397	0.130
FQ CC	11 (26.2)	12 (28.6)	8 (32.0)	0.20	0.903	0.043
FQ EOI	17 (40.5)^a^	0 (0.00)^b^	13 (52.0)^a^	26.99	< 0.001	0.498

*ARFID*  Avoidant/restrictive food intake disorder,* HC* Healthy controls,* AN*  Anorexia nervosa,* EDE-Q8*  Eating Disorder Examination-Questionnaire 8,* PHQ-9* Patient Healthy Questionnaire 9, *FQ* Family Questionnaire, (cutoff score)

Different superscripts denote group differences

Repeated measures analysis of variance (see Table 3) showed a statistically significant difference between parental depressive symptoms in patients with ARFID versus HC with large effect, *p* <.05, with parents of children with ARFID showing more depressive symptoms. Additionally, for emotional overinvolvement, parents of children with ARFID showed significantly higher scores than HC with large effect, *p* <.001. No other significant differences between ARFID and HC were observed. Parents of children with ARFID did not significantly differ from those with AN in all variables under study, *F*(4, 61) = 1.298, *p* =.281, with medium effect size (η_p_² = 0.078).

**Table 3:** Group differences in parental psychopathology and parental expressed emotion

	ARFID	HC	AN	ARFID vs. HC^a^			ARFID vs. AN^b^		
	*M ± SD*	*M ± SD*	*M ± SD*	*F*(df)	*p*	η_p_²	*F*(df)	*p*	η_p_²
EDE-Q8	1.43 *±* 1.50	1.40 *±* 1.46	1.40 *±* 1.60	0.14 (1, 41)	0.907	0.000	0.007 (2, 106)	0.997	0.000
PHQ-9	6.81 *±* 4.37	4.54 *±* 3.70	6.00 *±* 3.32	6.96 (1, 41)	0.012	0.145	3.632 (2, 106)	0.670	0.064
FQ CC	19.10 *±* 6.38	19.17 *±* 5.04	19.40 *±* 5.92	0.00 (1, 40)	0.950	0.000	0.035 (2, 105)	0.979	0.001
FQ EOI	25.33 *±* 5.82	18.52 *±* 3.79	27.20 *±* 5.38	37.41 (1, 41)	< 0.001	0.477	29.959 (2, 106)	0.384	0.361

*ARFID* Avoidant/restrictive food intake disorder, *HC* Healthy controls, *AN* Anorexia nervosa, *EDE-Q8* Eating Disorder Examination-Questionnaire 8, *PHQ-9* Patient Health Questionnaire 9, *FQ* Family Questionnaire, *CC* Critical comments, *EOI* =Emotional overinvolvement

^a^Repeated measures analysis of variance

^b^Analysis of covariance controlled for sex and age

### Associations between child and parental characteristics in ARFID

Correlation analyses showed a significantly positive association between parental depressive symptoms (PHQ-9) and emotional undereating (*p* <.001, large effect), satiety responsiveness, and slowness in eating (*p*s < 0.05, medium effect; CEBQ) and a significantly negative association with BMI-SDS as well as number of accepted foods, with medium effect size (*p*s < 0.05). For CC (FQ), significantly positive associations with emotional undereating (*p* <.001, large effect), satiety responsiveness, and fussiness (*p*s < 0.05, medium effect; CEBQ) emerged. For EOI (FQ), there were significantly positive associations with emotional undereating, satiety responsiveness, and slowness in eating (*p*s < 0.05, medium effect; CEBQ) and significantly negative associations with children’s BMI-SDS (*p* <.001) as well as number of accepted foods (*p* <.05), with medium effect size, see Table 4.

**Table 4:** Pearson correlation between child illness severity and symptomology and parental psychopathology and expressed emotion

	EDE-Q8 global mean score				PHQ-9 mean score			FQ CC subscale score				FQ EOI subscale score		
	*n*	*r*	*p*		*n*	*r*	*p*	*n*	*r*	*p*		*n*	*r*	*p*
BMI-SDS	109	0.039	0.689		109	− 0.191	0.047	108	− 0.004	0.965		109	− 0.337	< 0.001
No. of accepted foods	96	− 0.139	0.177		96	− 0.245	0.016	95	0.093	0.370		96	− 0.236	0.020
CEBQ SR score	107	0.022	0.825		107	0.223	0.021	106	0.205	0.035		107	0.310	0.001
CEBQ SE score	107	− 0.091	0.352		107	0.260	0.007	107	0.044	0.652		107	0.243	0.012
CEBQ EUE score	106	0.080	0.414		106	0.345	< 0.001	106	0.305	0.001		106	0.247	0.011
CEBQ FF score	107	0.022	0.820		107	0.136	0.163	107	0.210	0.030		107	0.150	0.123

*ARFID* Avoidant/restrictive food intake disorder, *BMI* Body mass index, *SDS* Standard deviation score, *CEBQ* Child Eating Behavior Questionnaire, *SR* Satiety Responsiveness, *SE* Slowness in Eating, *EUE* Emotional Undereating, *FF* Food Fussiness, *EDE-Q8* Eating Disorder Examination–Questionnaire 8, *PHQ-9* Patient Health Questionnaire 9, *FQ* Family Questionnaire, *CC* Critical Comments, *EOI* Emotional Overinvolvement

## Discussion

As one of the few studies on investigating parental psychopathology and parental expressed emotion in children and adolescents with ARFID, this study showed that parents of families having a child with ARFID (*n* = 42) reported significantly more depressive symptoms and emotional overinvolvement than parents of healthy children (*n* = 42) matched for age and sex, while no significant difference emerged in relation to parents having a child with anorexia nervosa (AN, *n* = 25). Children’s lower standardized BMI, lower number of accepted foods, and higher avoidant/restrictive eating behaviors were significantly related to higher parental depressive symptoms, critical comments, and emotional overinvolvement, but unrelated to parental eating disorder psychopathology comprising restraint, eating concern, shape concern and weight concern, highlighting the relational nature of parental factors and illness characteristics in restrictive eating disorders.

Consistent with limited available evidence on parental psychopathology in children aged < 3 years with ARFID or a similar feeding disorder [32, 35–37, 39] the present study found significantly higher levels of parental self-reported depressive symptoms in ARFID compared to healthy controls (HC). Descriptively, one quarter of parents having a child with ARFID and one fifth of parents having a child with AN exceeded the clinical cut-off score of the PHQ-9, reflecting a considerable psychological burden characterized by depressive symptoms in parents of children with restrictive ED. This result is in line with elevated parental depressive symptoms (20–38%) in hospitalized adolescents with AN [23, 24, 57]. In contrast to previous findings on parental eating disorder psychopathology assessed by self-report in ARFID or related feeding disorder [35, 36, 38, 39] as well as in AN or other eating disorders [58, 59], no significant group differences emerged for parental eating disorder psychopathology. Descriptively, 7% of parents having a child with ARFID exceeded the 90 th percentile on the EDE-Q8, which was less often than parents of families from the HC (9%) or having a child with AN (12%), indicating parental eating disorder psychopathology related to restraint, eating concern, shape concern and weight concern to be rather irrelevant in ARFID.

In terms of expressed emotion, with evidence only being available for families with AN so far, this study identified 40% of families with a child affected by ARFID exceeding clinical cutoffs for emotional overinvolvement, which was significantly higher compared to HC (0%), but not different from AN (52%). In line with the present results, high levels of emotional overinvolvement in caregivers of a child with an eating disorder, mainly AN, as assessed via the FQ have previously been found (27–89%; see review by Anastasiadou et al. [19]). Given that emotional overinvolvement describes overprotectiveness, overconcern, and overidentification, parents of children with ARFID and AN—both severe and potentially life-threatening disorders—plausibly show higher involvement in children’s life than parents of children without a restrictive feeding disorder or eating disorder [60]. While increased emotional overinvolvement may indicate an effort to increase the child’s food intake—particularly since food intake can be seen as a potentially controllable behavior and, mostly in younger children, as a responsibility of the parents—it is also important to consider the extent to which parental emotional overinvolvement may have influenced the child’s avoidant-restrictive food intake behavior. Compared to emotional overinvolvement, criticism was lower in families with a child with ARFID or AN, consistent with previous findings in families with children having an eating disorder [19]. In the present study, 26% of parents having a child with ARFID exceeded clinical cutoffs for critical comments, with a nonsignificant different level compared to HC (29%) and AN group (32%), which indicates that critical comments may be common in children and adolescents independent from an existing disorder. In the context of restrictive eating disorders, our results suggest that parental emotional overinvolvement is more prominent than critical comments [see also 57]. This may reflect parents’ heightened concern or anxiety, leading to overprotectiveness rather than overt critique. Notably, the Family Questionnaire only measures general critical comments, but no food-specific criticism. Thus, parental behaviors, such as pressure to eat—potentially perceived as critical—may not have been adequately captured, but still play a role in the dynamics of restrictive eating disorders [61].

Regarding associations between parental psychopathology and children’s characteristics related to avoidant-restrictive food intake, this study uniquely showed that parental depressive symptoms and emotional overinvolvement were adversely related to child symptoms, which may mirror the group differences in parental characteristics described above. In line with previous findings [10, 22–24, 35], parental depressive symptoms and more emotional overinvolvement were associated with lower BMI-SDS, satiety responsiveness, number of accepted foods, and more slowness in eating and emotional undereating, indicating elevated illness severity in children with AN and ARFID or related feeding disorder to be associated with greater parental psychological distress and higher levels of emotional involvement. Importantly, in this study, more food fussiness was associated with more critical comments only, suggesting similarity with former findings on positive associations between food fussiness in children and parental pressure to eat, which may be seen as critical parental expression [61]. Lacking associations between parental eating disorder psychopathology - specifically restraint, eating concern and shape- and weight-related concerns - and child characteristics may be related to the composition of the sample including both adolescents showing traditional eating disorder psychopathology and younger children without weight and shape concern. This heterogeneity may have leveled out associations previously observed in ARFID and AN [35, 39, 58, 59].

Strengths of the present study include an interview-based diagnosis, the use of well-established questionnaires and objective anthropometrics, and particularly the individual matching between participants with ARFID and participants without feeding disorder or eating disorder. Still, the cross-sectional nature of the presented study does not allow causal interpretation of the results. Given the large age range of study participants and age differences between the ARFID and AN group, potentially confounding factors apart from age may have an influence on parental psychopathology and expressed emotion, such as illness duration. A general limitation of the study lies in the broad age range of participants (0–17 years), which made it necessary to rely on different informants (parents vs. adolescents) depending on age and feasibility. This applies in particular to the assessment of food variety, which was based on a non-standardized food list completed either by parents or adolescents. While the list has been used in previous ARFID studies, variation in reporting sources may have affected consistency. Standardized, age-appropriate tools are needed to improve comparability across developmental stages. Another limitation concerns the operationalization of expressed emotion. While expressed emotion is conceptually a multidimensional construct comprising five components (criticism, hostility, emotional overinvolvement, warmth, and positive remarks), the present study assessed only criticism and emotional overinvolvement, as measured by the Family Questionnaire. As such, our findings reflect a partial representation of the broader expressed emotion construct, and future research may benefit from more comprehensive assessments that include all dimensions of expressed emotion. Moreover, ratings of expressed emotion were obtained exclusively from mothers, and paternal expressed emotion was not assessed. This limits the generalizability of our results to the maternal perspective and may overlook potentially important contributions of paternal expressed emotion. Future research should aim to include both parents and all dimensions of expressed emotion to capture a more comprehensive picture of family emotional climate. Finally, we did not assess ARFID symptomatology and comorbidities in parents, nor comorbidities in participants, which should be considered when interpreting the results. Future studies are warranted to address these characteristics in order to provide a more comprehensive understanding of familial transmission and potential intergenerational patterns associated with ARFID.

## Conclusion

Although causality cannot be inferred due to cross-sectional design, the study findings offer a possible transfer of concepts around parental psychopathology and expressed emotion in feeding disorders and AN to ARFID. Because expressed emotion has been shown to be a stable and accurate predictor of relapse in various mental disorders, including schizophrenia and affective disorders [17] as well as AN [15, 25, 62], future longitudinal studies may focus on the role of expressed emotion on the long-term outcome of ARFID. Examining potential mediators on the association between parental depressive symptoms and expressed emotion with child symptoms, such as parental feeding practices and general parenting, would be highly valuable to identify specific targets for interventions. Clinically, considering the present results on family factors in ARFID, family-based treatment approaches [63, 64] in ARFID should prioritize addressing emotional overinvolvement and parental depressive symptoms if deemed necessary.

## Electronic supplementary material

Supplementary Material 1

## Data Availability

The data are available from the senior author upon reasonable request.
