# Supplementary material for: Parental psychopathology and expressed emotion in children with avoidant/restrictive food intake disorder
Source: Child Adolesc Psychiatry Ment Health. 2025 Jun 6;19:66. doi: 10.1186/s13034-025-00929-x (PMC12142924; doi:10.1186/s13034-025-00929-x)
Supplement: Supplementary file 1 — Supplementary Material 1 [file 13034_2025_929_MOESM1_ESM.docx]

**Supplement**

**Table S1**

*Pearson Correlation between Parental Psychopathology and Expressed Emotions in the Total Sample*

|  | EDE-Q8 | | | PHQ-9 | | | FQ-CC | | | FQ-EOI | | |
| --- | --- | --- | --- | --- | --- | --- | --- | --- | --- | --- | --- | --- |
|  | *n* | *r* | *p* | *n* | *r* | *p* | *n* | *r* | *p* | *n* | *r* | *p* |
| EDE-Q8 | 109 | 1 | - |  |  |  |  |  |  |  |  |  |
| PHQ-9 | 109 | .173 | .072 | 109 | 1 | - |  |  |  |  |  |  |
| FQ-CC | 108 | .116 | .233 | 109 | .417 | <.001 | 108 | 1 | - |  |  |  |
| FQ-EOI | 109 | .242 | .011 | 109 | .588 | <.001 | 108 | .408 | <.001 | 109 | 1 | - |

*Note.* EDE-Q8 = Eating Disorder Examination–Questionnaire 8, PHQ-9 = Patient Health Questionnaire 9, FQ = Family Questionnaire, CC = Critical Comments, EOI = Emotional Overinvolvement.

**Table S2**

*Illness Severity and Symptomology in the Total Sample*

|  | *N* | *M* | *SD* | range |
| --- | --- | --- | --- | --- |
| BMI-SDS | 109 | -1.06 | 1.14 | -3.8 – 2.2 |
| No. of accepted foods | 96 | 93.77 | 56.82 | 2.0 – 235.0 |
| CEBQ |  |  |  |  |
| Satiety responsiveness | 107 | 2.20 | 0.80 | 0.40 – 4.0 |
| Slowness in eating | 107 | 2.01 | 0.85 | 0.50 – 4.0 |
| Emotional undereating | 106 | 1.89 | 0.99 | 0.00 – 4.0 |
| Food fussiness | 107 | 2.20 | 1.06 | 0.00 – 4.0 |

*Note.* ARFID = Avoidant/restrictive food intake disorder; BMI = body mass index; SDS = standard deviation score; CEBQ = Child Eating Behavior Questionnaire.

**Table S3**

*Illness Severity and Symptomology in those with ARFID*

|  | *N* | *M* | *SD* | range |
| --- | --- | --- | --- | --- |
| BMI-SDS | 42 | -1.55 | 1.04 | -3.7 – 1.08 |
| No. of accepted foods | 41 | 63.66 | 43.96 | 2.0 – 179.0 |
| CEBQ |  |  |  |  |
| Satiety responsiveness | 41 | 2.59 | 0.63 | 1.2 – 4.0 |
| Slowness in eating | 40 | 2.14 | 0.89 | 0.5 – 4.0 |
| Emotional undereating | 40 | 2.27 | 1.00 | 0.3 – 4.0 |
| Food fussiness | 40 | 2.86 | 0.83 | 1.0 – 4.0 |

*Note.* ARFID = Avoidant/restrictive food intake disorder; BMI = body mass index; SDS = standard deviation score; CEBQ = Child Eating Behavior Questionnaire.
